# Supplementary material for: Accounting for skill in trend, variability, and autocorrelation facilitates better multi-model projections: Application to the AMOC and temperature time series
Source: PLoS One. 2019 Apr 10;14(4):e0214535. doi: 10.1371/journal.pone.0214535 (PMC6457553; doi:10.1371/journal.pone.0214535)
Supplement: S1 File — (PDF) [file pone.0214535.s001.pdf]

Supporting Information for "Accounting for skill in trend, variability, and autocorrelation facilitates better multi-model projections: application to the AMOC and temperature time series"

Projections accounting for model trend and variability skill

Roman Olson[1,2,3], Soon-Il An[1], Yanan Fan[4] and Jason P. Evans[5]

[1]Department of Atmospheric Sciences, Yonsei University, Seoul, South Korea

[2]Center for Climate Physics, Institute for Basic Science, Busan, South Korea

[3]Pusan National University, Busan, South Korea

[4]School of Mathematics and Statistics, UNSW Australia, Sydney, NSW, 2052, Australia

[5]Climate Change Research Centre and ARC Centre for Excellence in Climate Extremes, UNSW Australia, Sydney, NSW, Australia

Contents of this file

Texts A and B
Figs A to T
Tables A to B

Introduction

Supporting Texts contains information on details of the statistical methodology. Text A provides technical details on weighting trend models, while Text B discusses the implementation of the variability model weighting.

Text A. Technical details on weighting the trend models

For the Monte Carlo integration to get trend submodel weights, we use 100,000 samples for all experiments except "trend" Korea_temp, AMOCIndex and AMOCIndex_obs which use 1,000,000 samples. The real-case Korean temperature projections also use 1,000,000 samples. When we repeat Korea_temp experiments with a different number of samples (1,000,000 for "trend+var" and 100,000 for "trend"), the performance metrics for these experiments are virtually identical. This suggests that 100,000 samples are enough to reasonably estimate method performance. We employ uniform priors for σ on [0, 5], and for ρ on [-1, 1] for the AR1 properties of the internal variability during the trend weighting in all experiments.

Text B. Implementation of the variability model weighting

For the Monte Carlo integration to get variability submodel weights, we use 10,000 samples for all relevant experiments. The real-case Korean temperature projections use 100,000 samples. Since the results for the performance metrics are virtually identical for the longer Korea_temp “trend+var” experiment described in Text A (which uses 100,000 samples for variability weights estimation), we deduce that 10,000 samples is a reasonable number. When sampling $\boldsymbol{\theta}_{\boldsymbol{y}}^{\boldsymbol{V}}$ we set autocorrelations with absolute values of 0.999 or higher to ±0.999 for numerical stability reasons. Likewise, we restrict ourselves to positive innovation standard deviations by setting all values below 0.01×min($\breve{\boldsymbol{\sigma}}_M$) to 0.01×min($\breve{\boldsymbol{\sigma}}_M$). Here $\breve{\boldsymbol{\sigma}}_M$ is a vector of all standard deviation summary statistics from all dynamical variability models: $\breve{\boldsymbol{\sigma}}_{\boldsymbol{M}} = (\breve{\sigma}_{M,1}, \dots, \breve{\sigma}_{M,k})$.

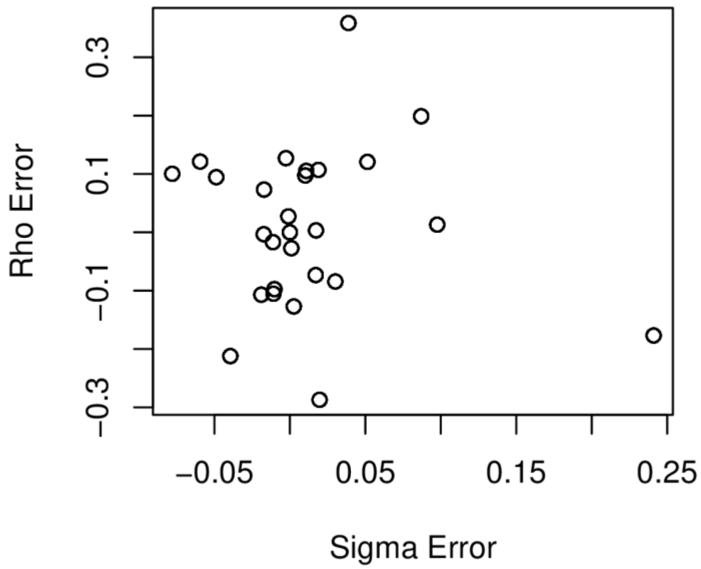

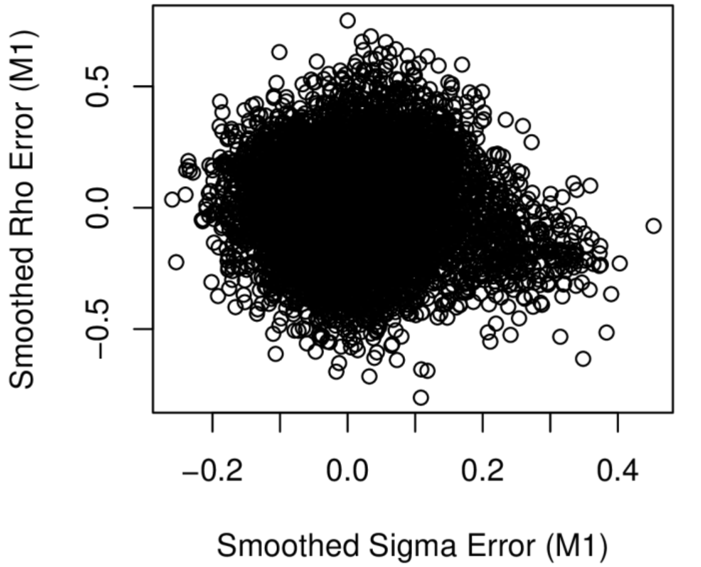

Fig A. (top) 26 samples of $f\boldsymbol{\varepsilon}^V = f(\varepsilon_\sigma , \varepsilon_\rho)$ provided by next-closest model differences for the Winter_SST experiment, together with the (0,0) sample, (bottom) samples augmented by samples from the bivariate normal distribution with standard deviations set to 1/5 of the initial sample ranges, used in weighting the 1st variability model.

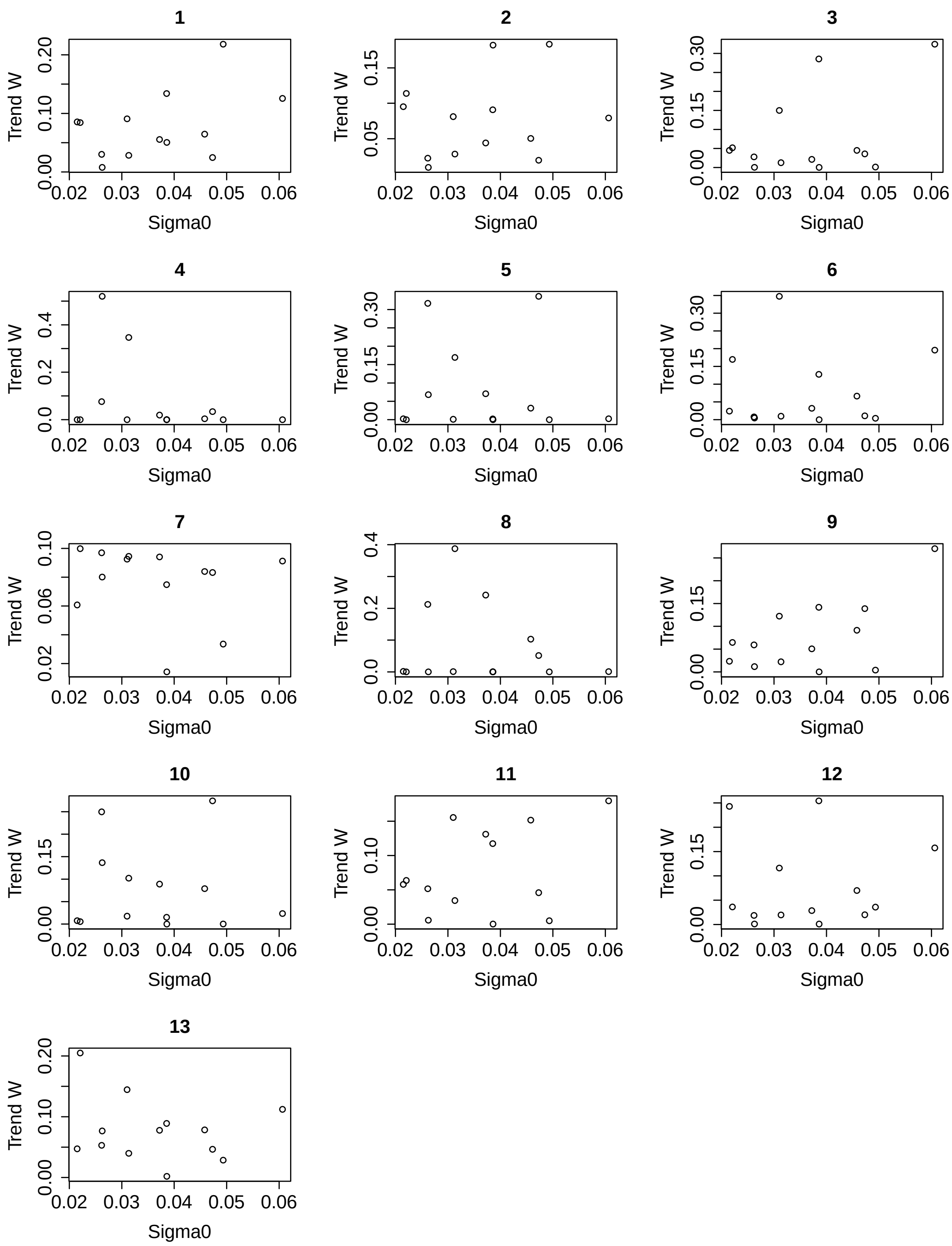

Fig B. Correlation between normalized innovation standard deviation summary statistics and trend weights $p(M_{T,i})$ for the AMOC experiment. Each panel corresponds to a different "true" model used as pseudo-observations.

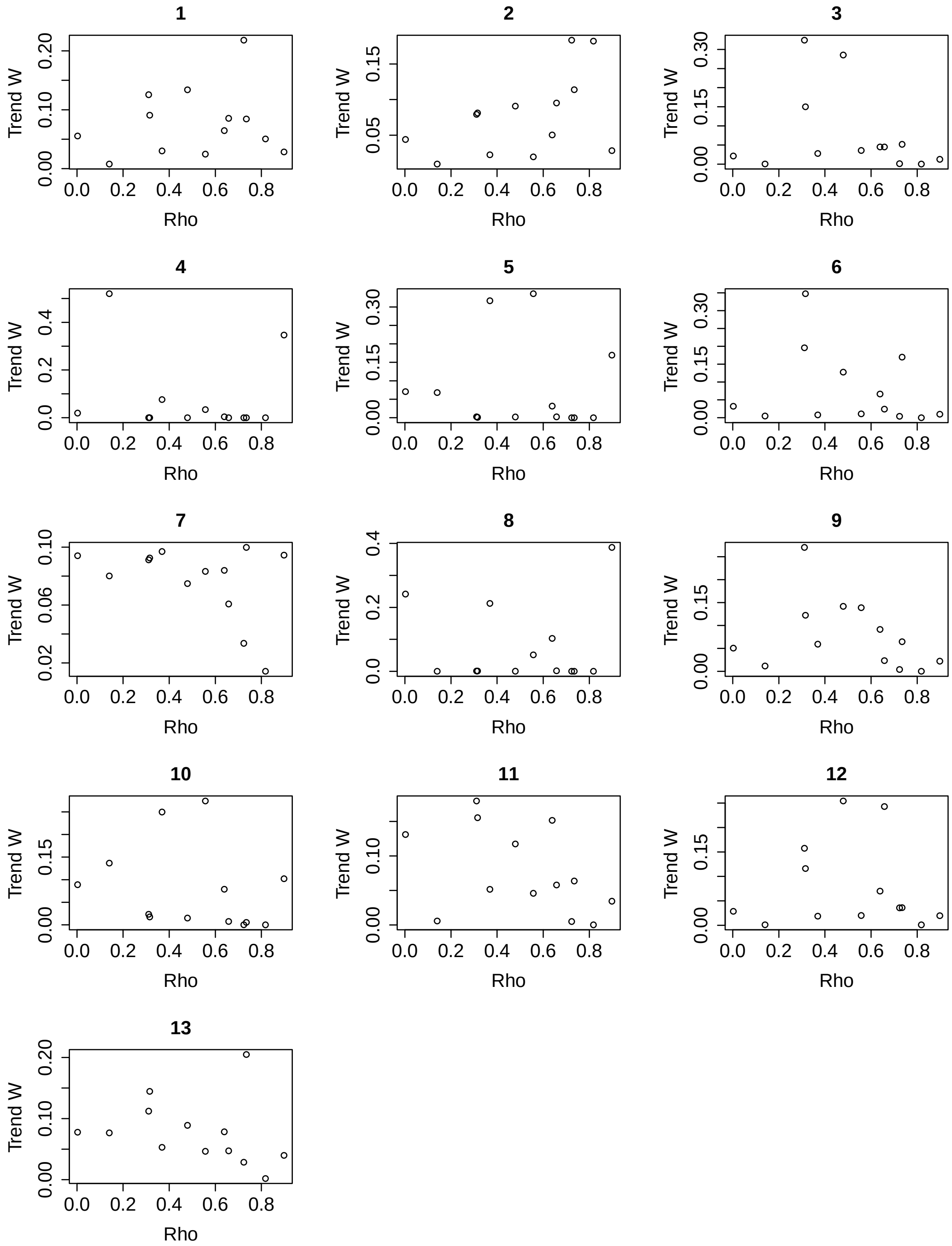

Fig C. Correlation between autocorrelation summary statistics and trend weights $p(M_{T,i})$ for the AMOC experiment. Each panel corresponds to a different "true" model used as pseudo-observations.

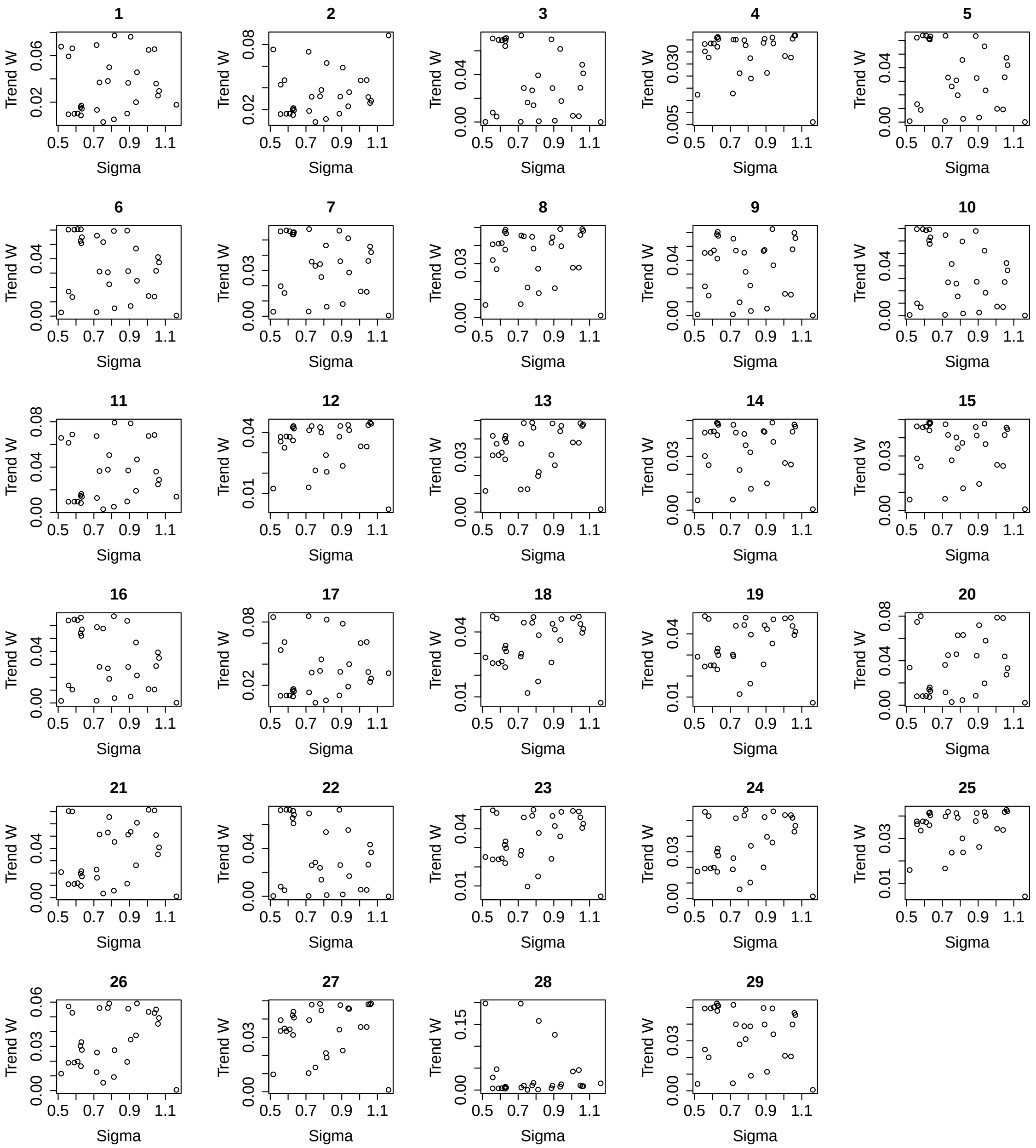

Fig D. Same as Fig B, but for un-normalized standard deviation [K] and the Korea_temp experiment.

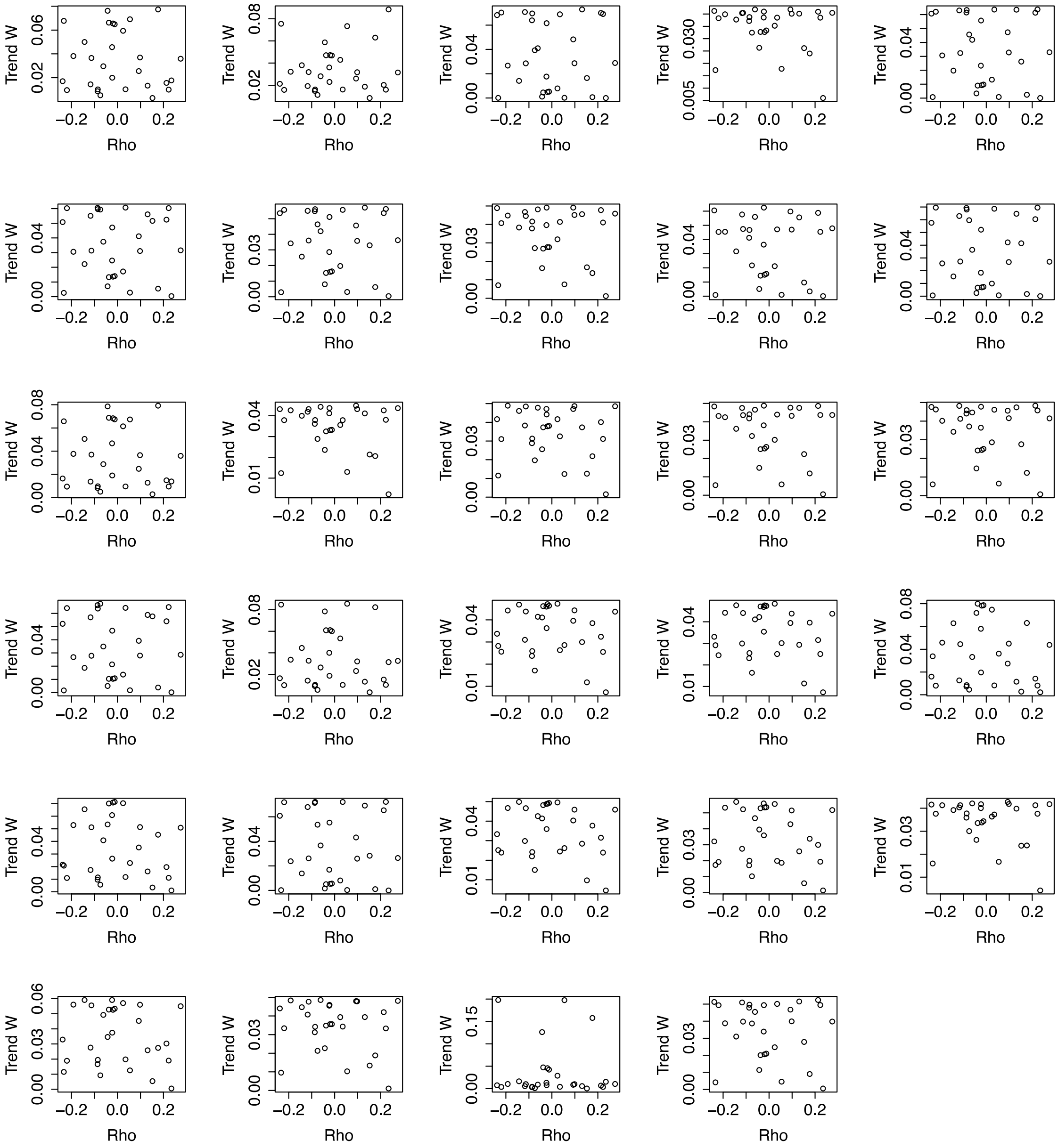

Fig E. Same as Fig C, but for the Korea_temp experiment.

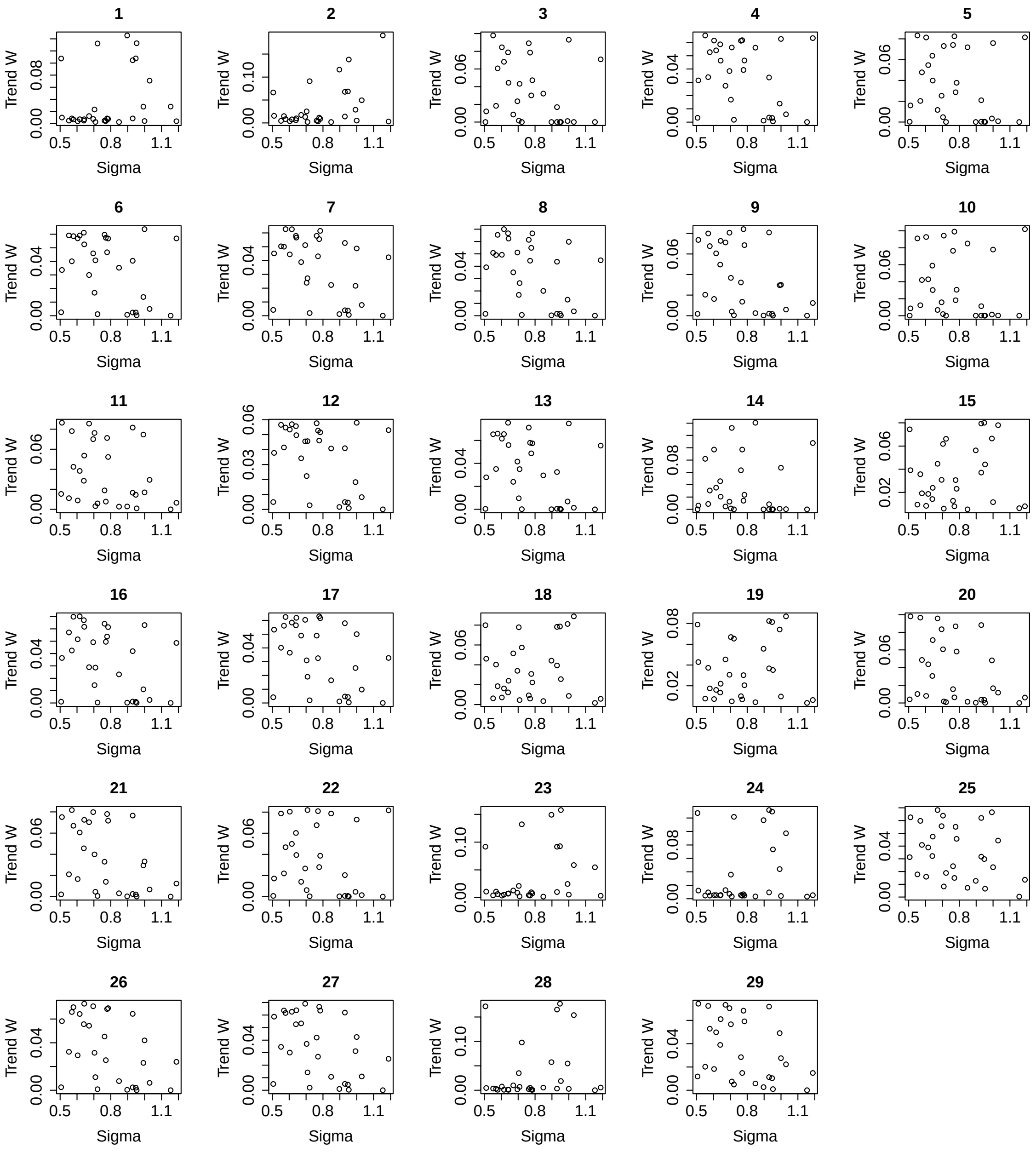

Fig F. Same as Fig B, but for un-normalized standard deviation [K] and the Korea_temp_long experiment.

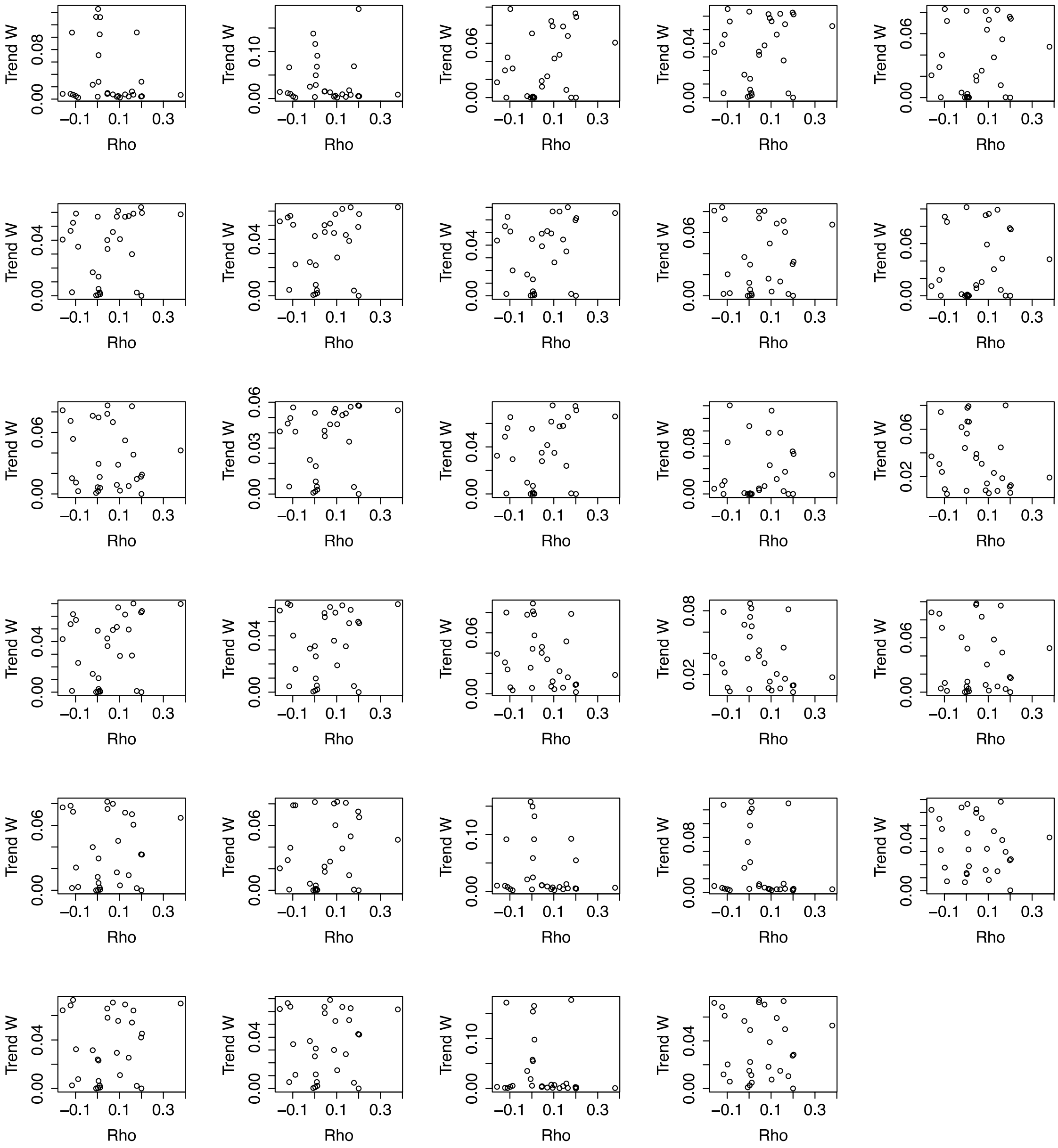

Fig G. Same as Fig C, but for the Korea_temp_long experiment.

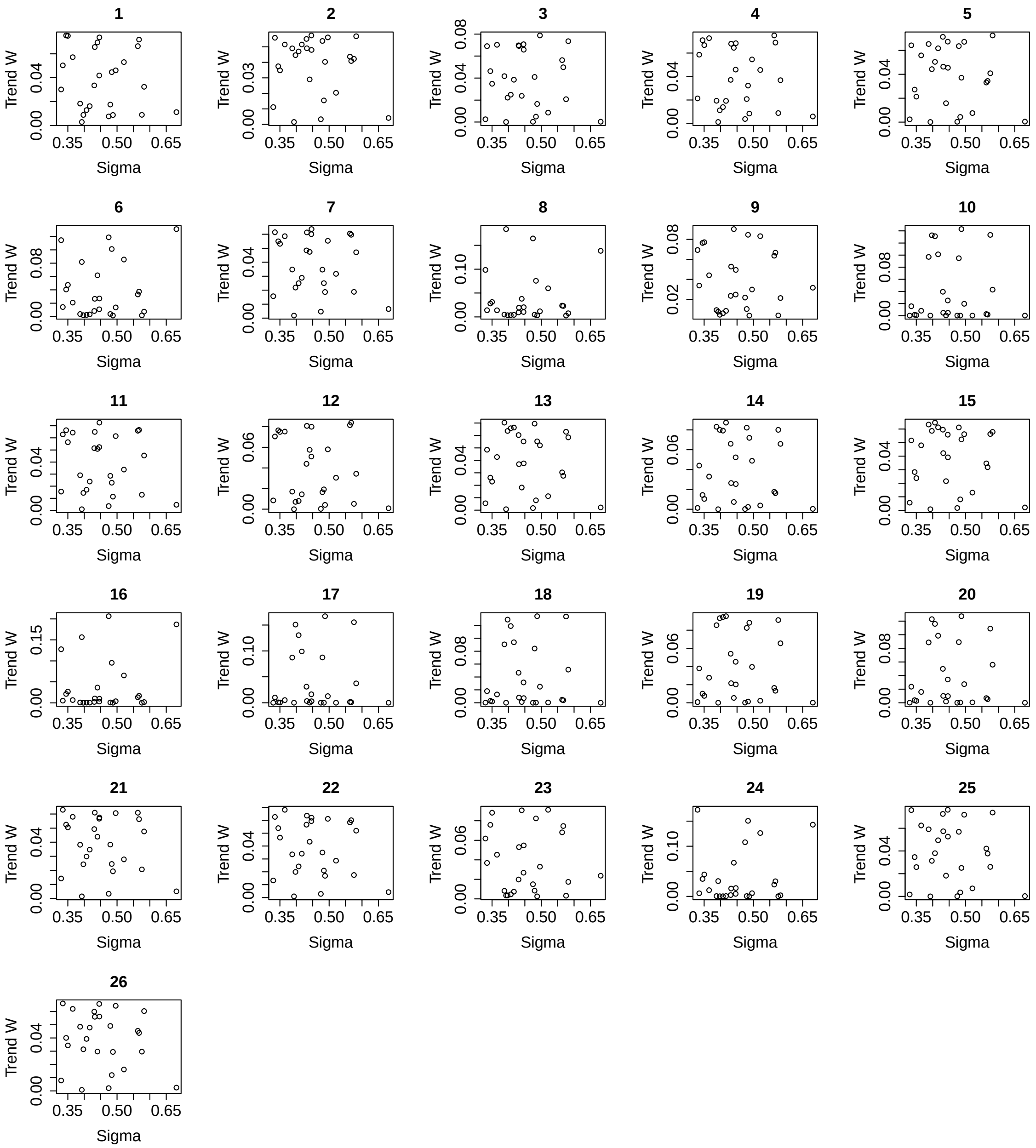

Fig H. Same as Fig B, but for un-normalized standard deviation [K] and the Winter_SST experiment.

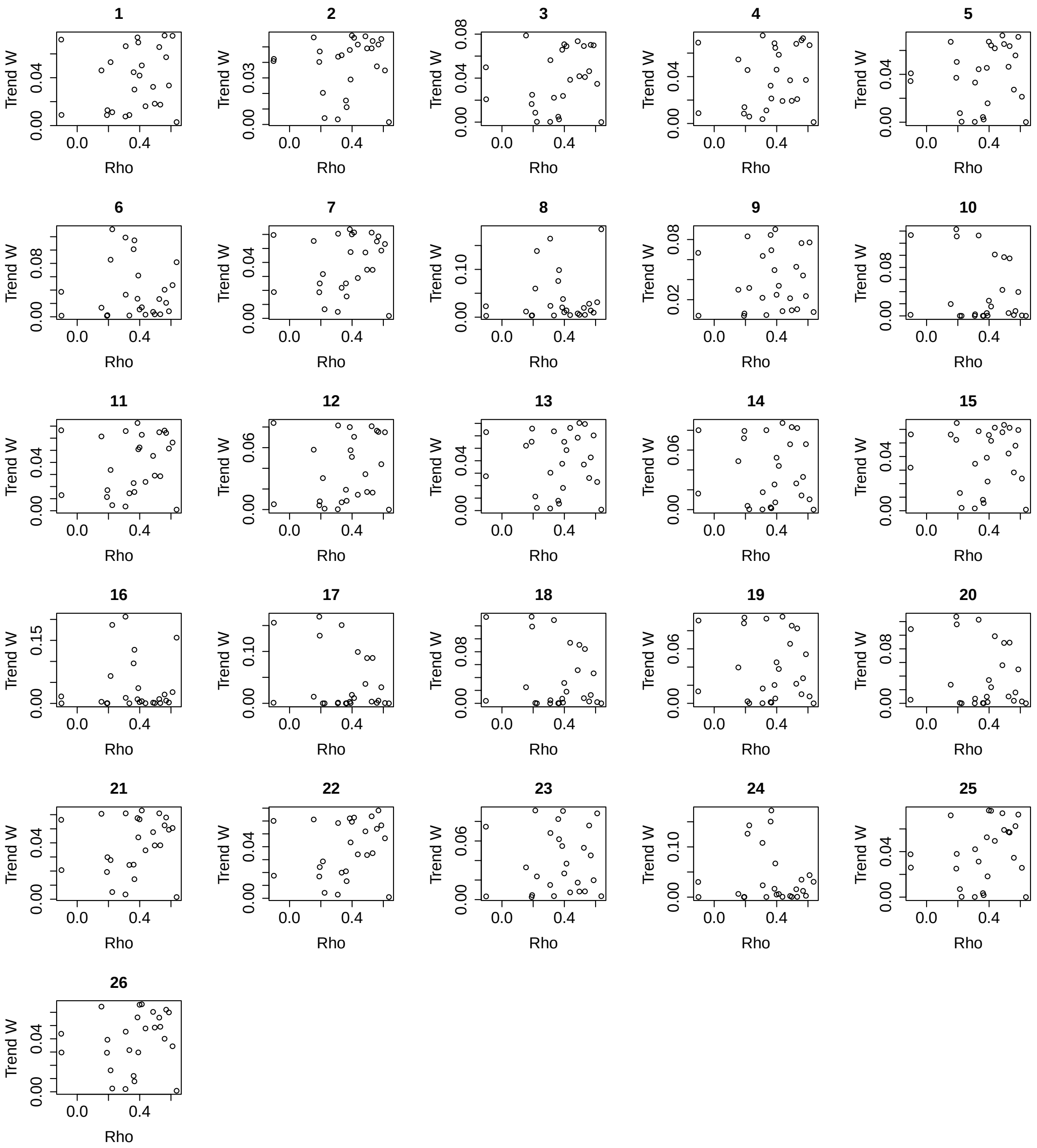

Fig I. Same as Fig C, but for the Winter_SST experiment.

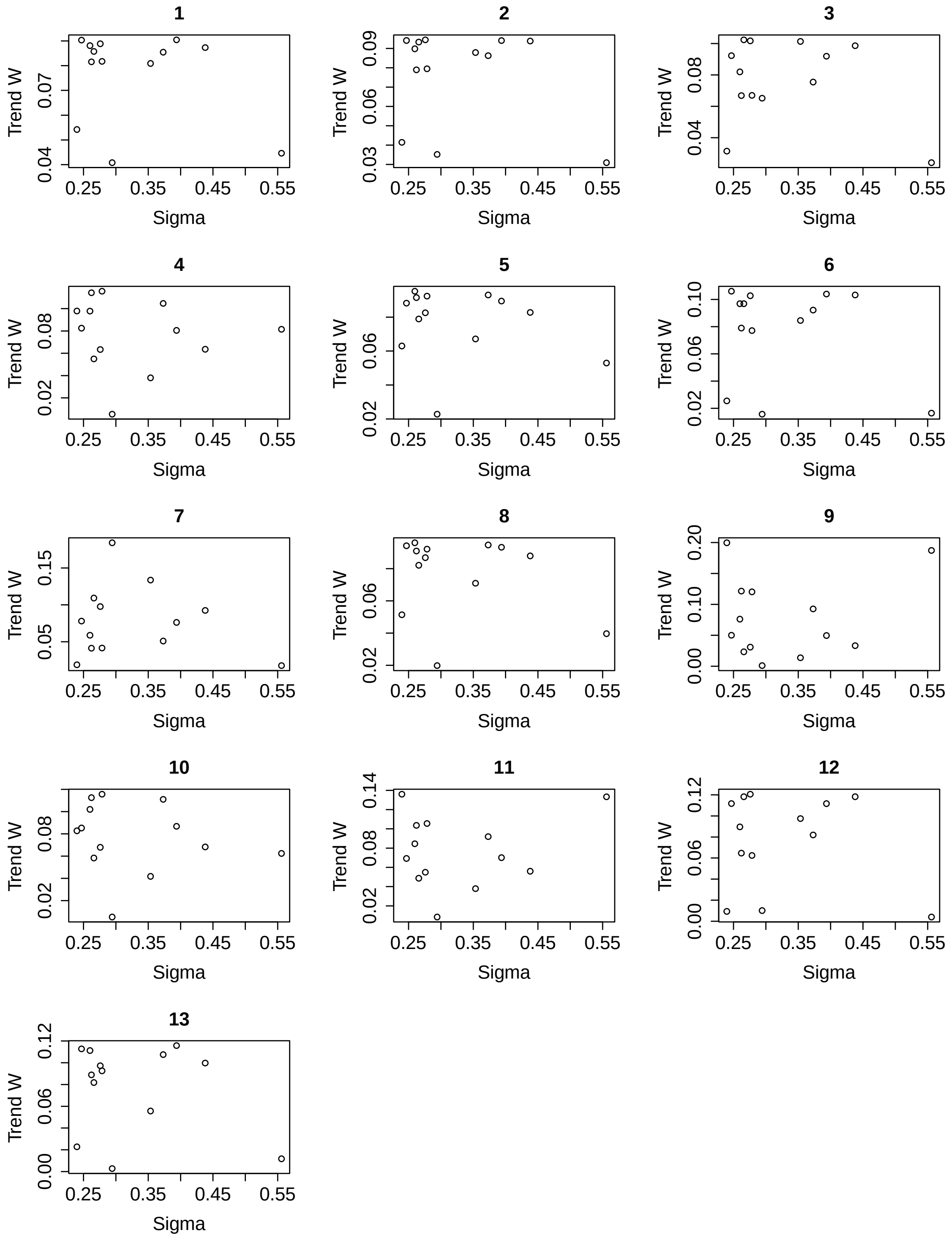

Fig J. Same as Fig B, but for un-normalized standard deviation [K] and the AMOCIndex experiment.

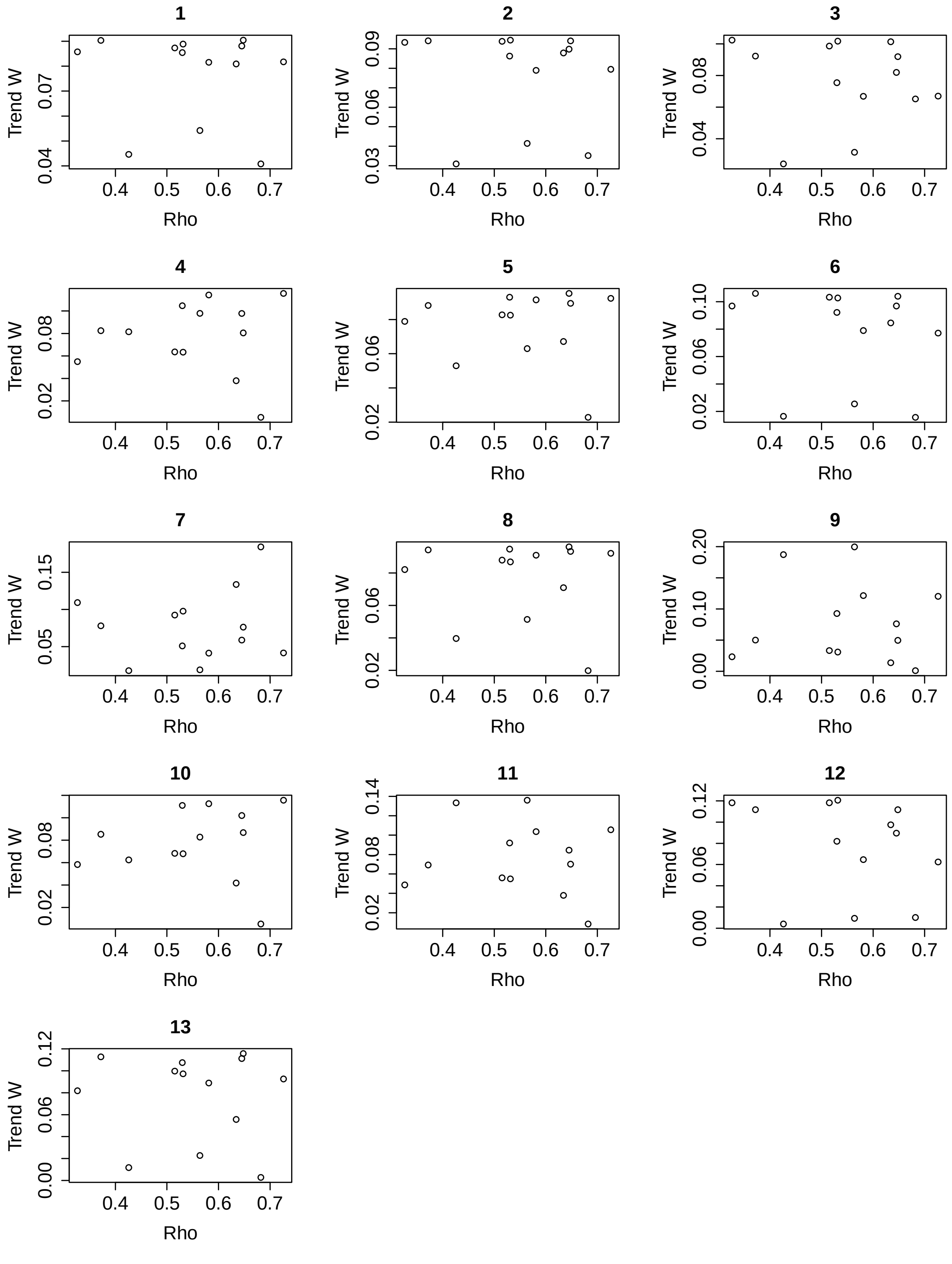

Fig K. Same as Fig C, but for the AMOCIndex experiment.

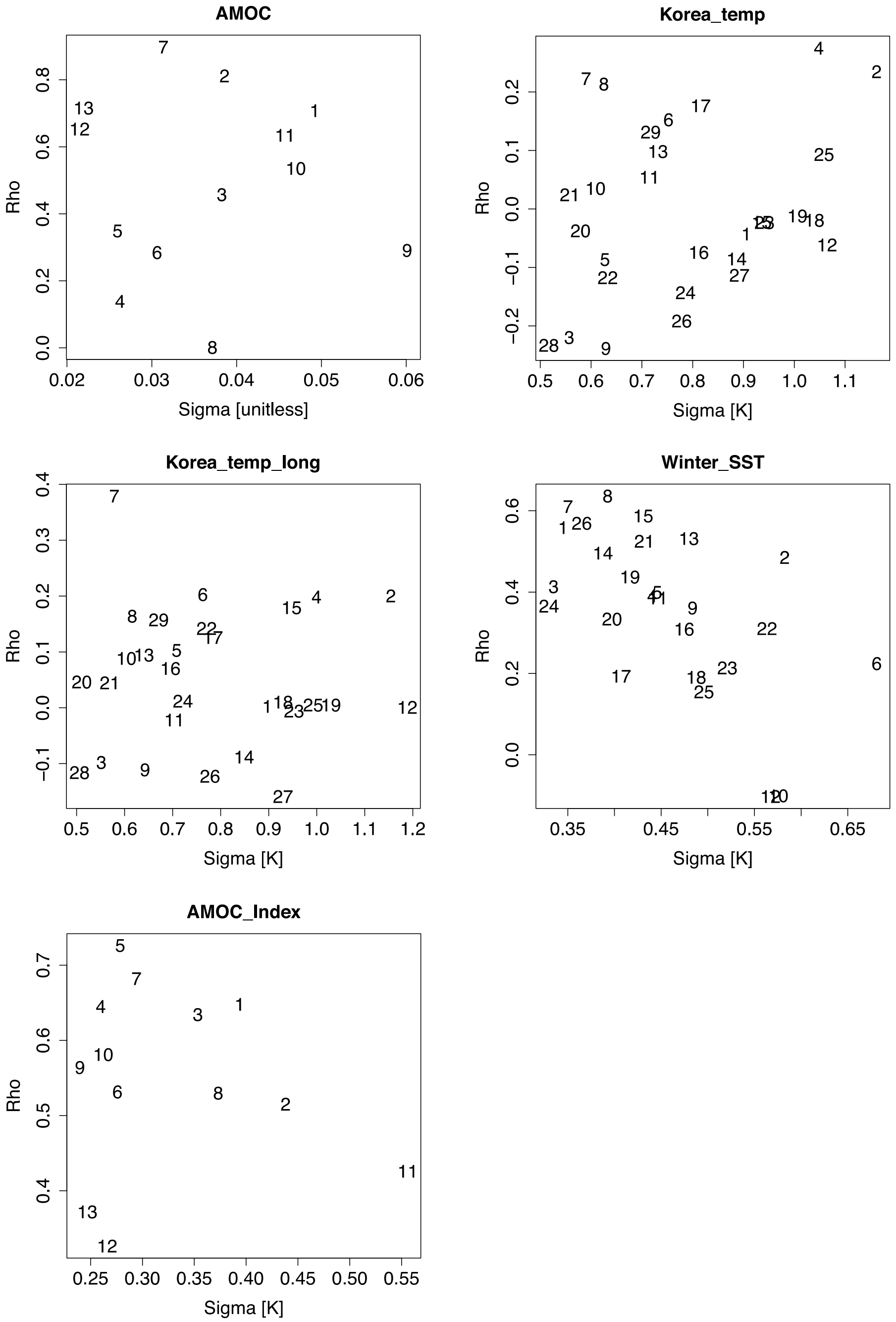

Fig L: Similar to Fig. 4, but with model numbers shown instead of future climate changes. For model numbers in AMOC and AMOC_Index experiments see previous work [1]. For the Korea_temp and Korea_temp_long experiments see Table A. For Winter_SST experiment see Table B.

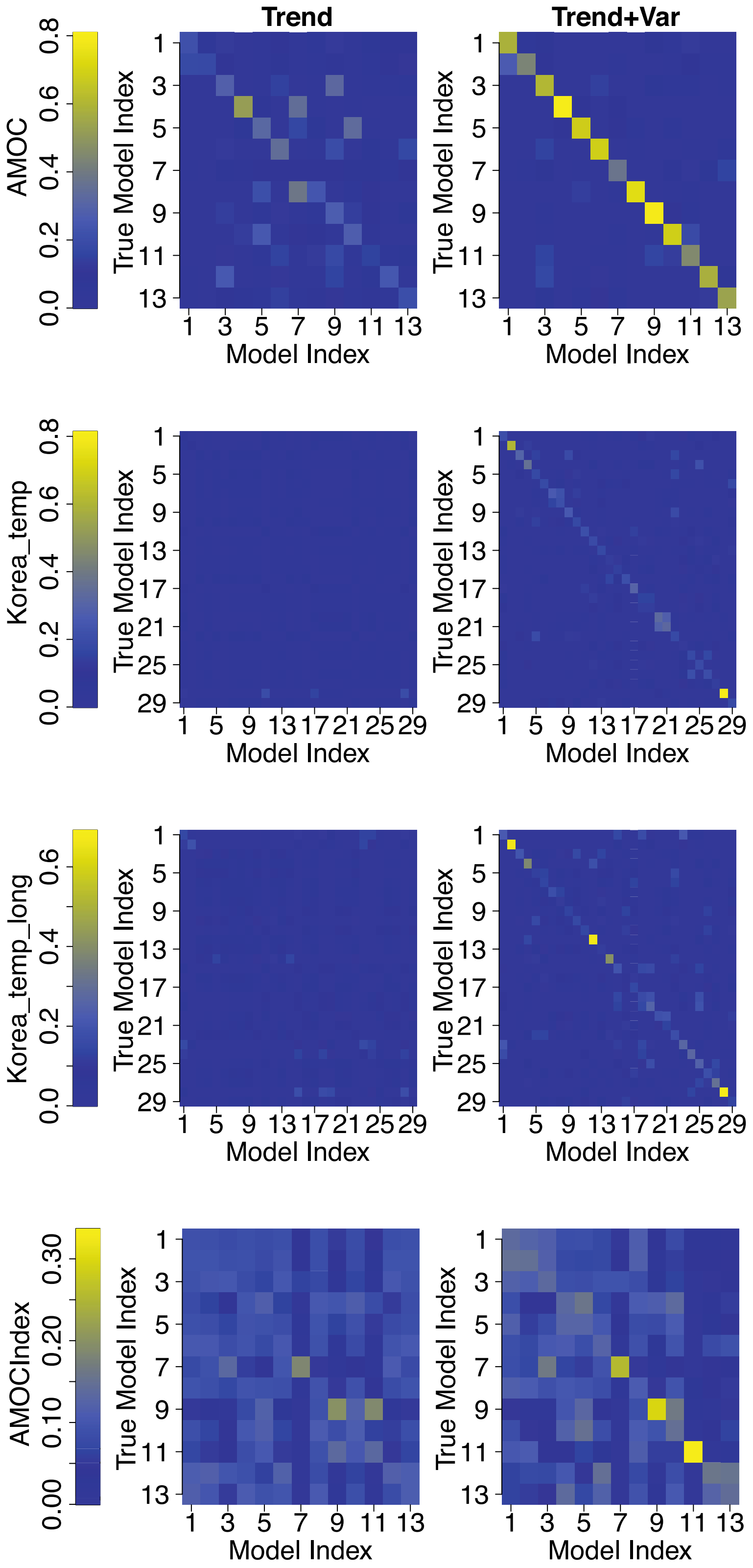

Fig M. Similar to Fig 5, but for the rest of one-at-a-time cross-validation experiments.

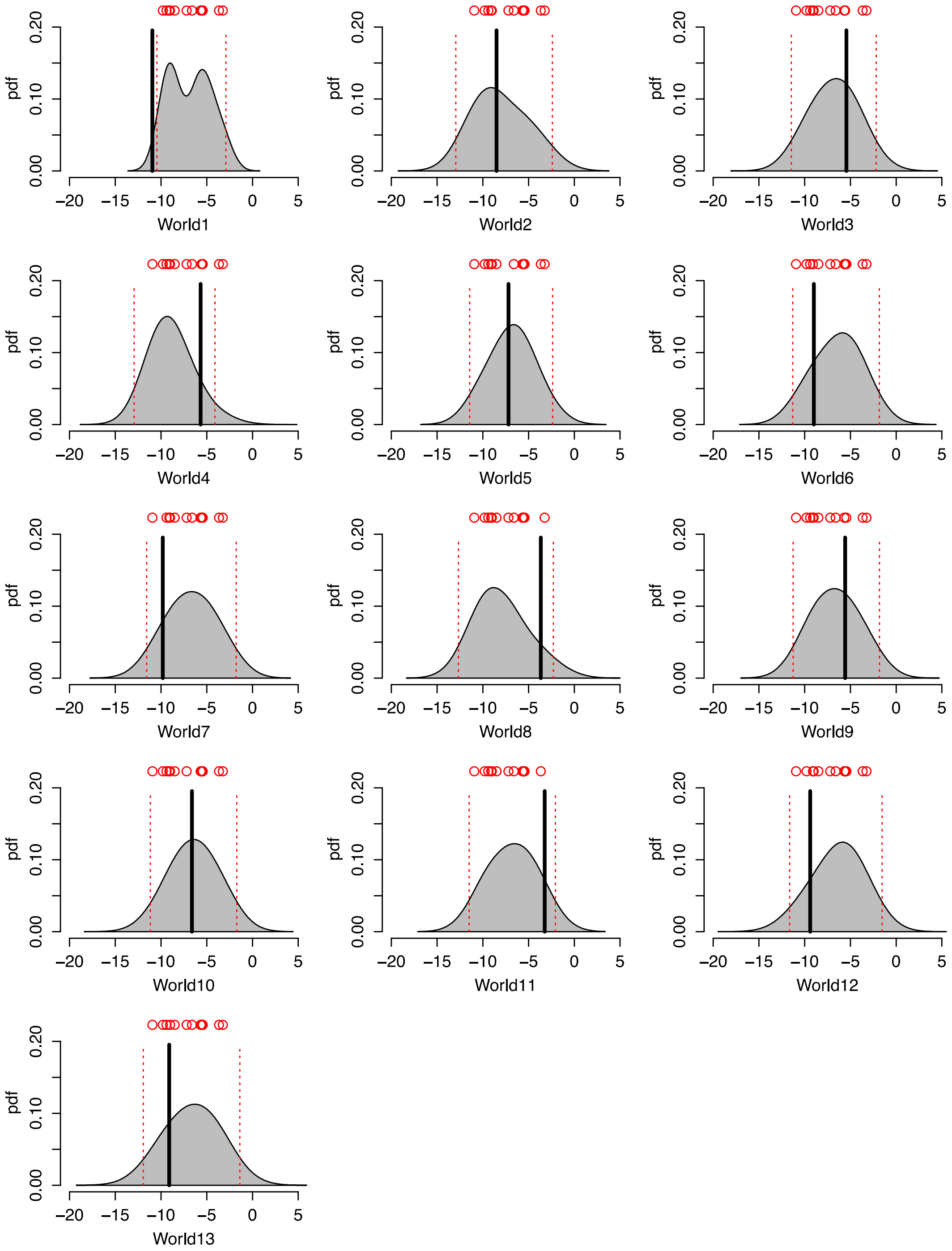

Fig N. Probabilistic projections for AMOC change from 1960-1999 to 2060-2099 [Sv] under the RCP8.5 emissions scenario for the "trend" AMOC cross-validation experiment. Subplots differ in the assumed "true" model. Red circles are deterministic projections from each model, red dotted lines are 90% posterior credible intervals. Black lines are changes from the "true" models.

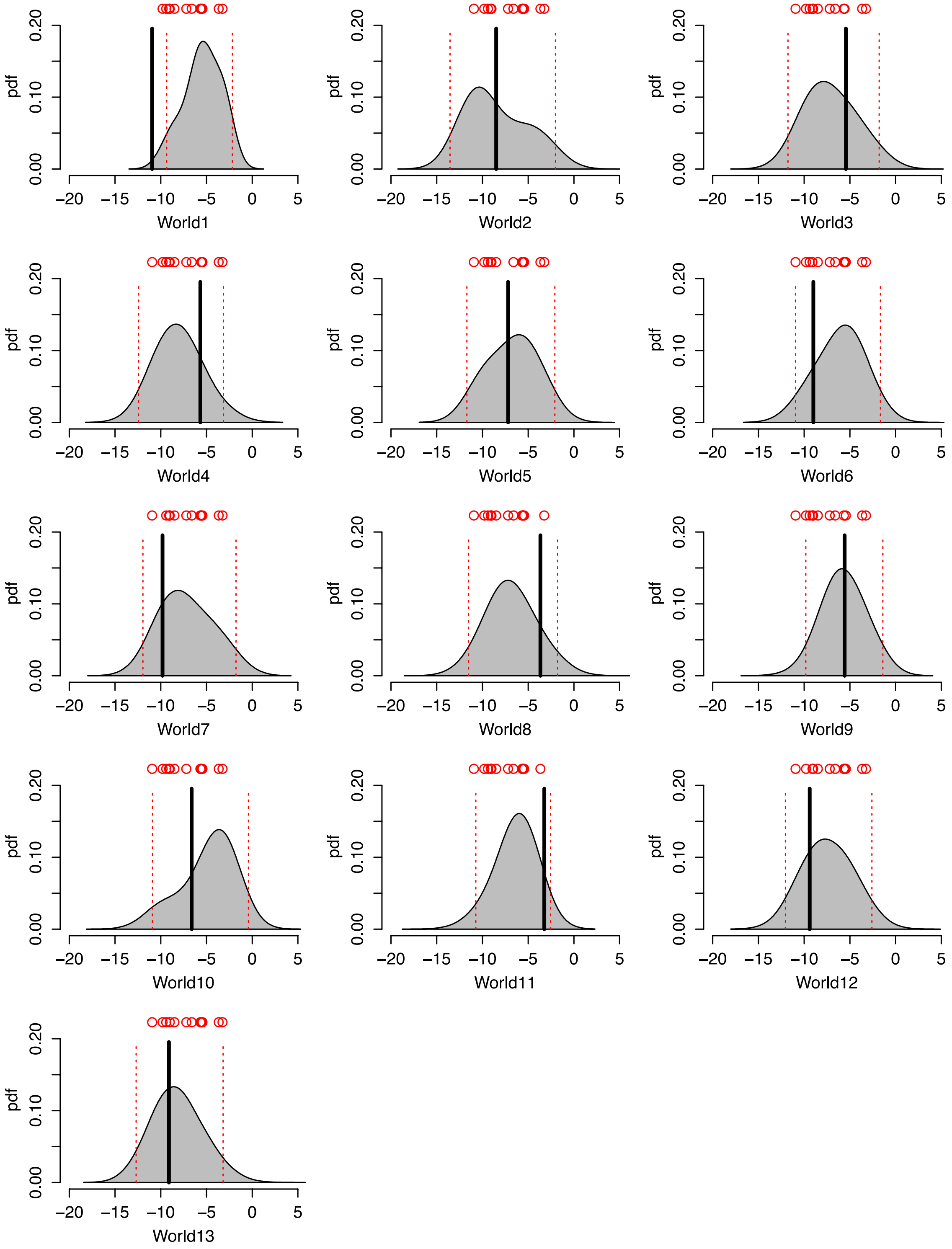

Fig O. Similar to Fig M, but for the AMOC “trend+var” experiment.

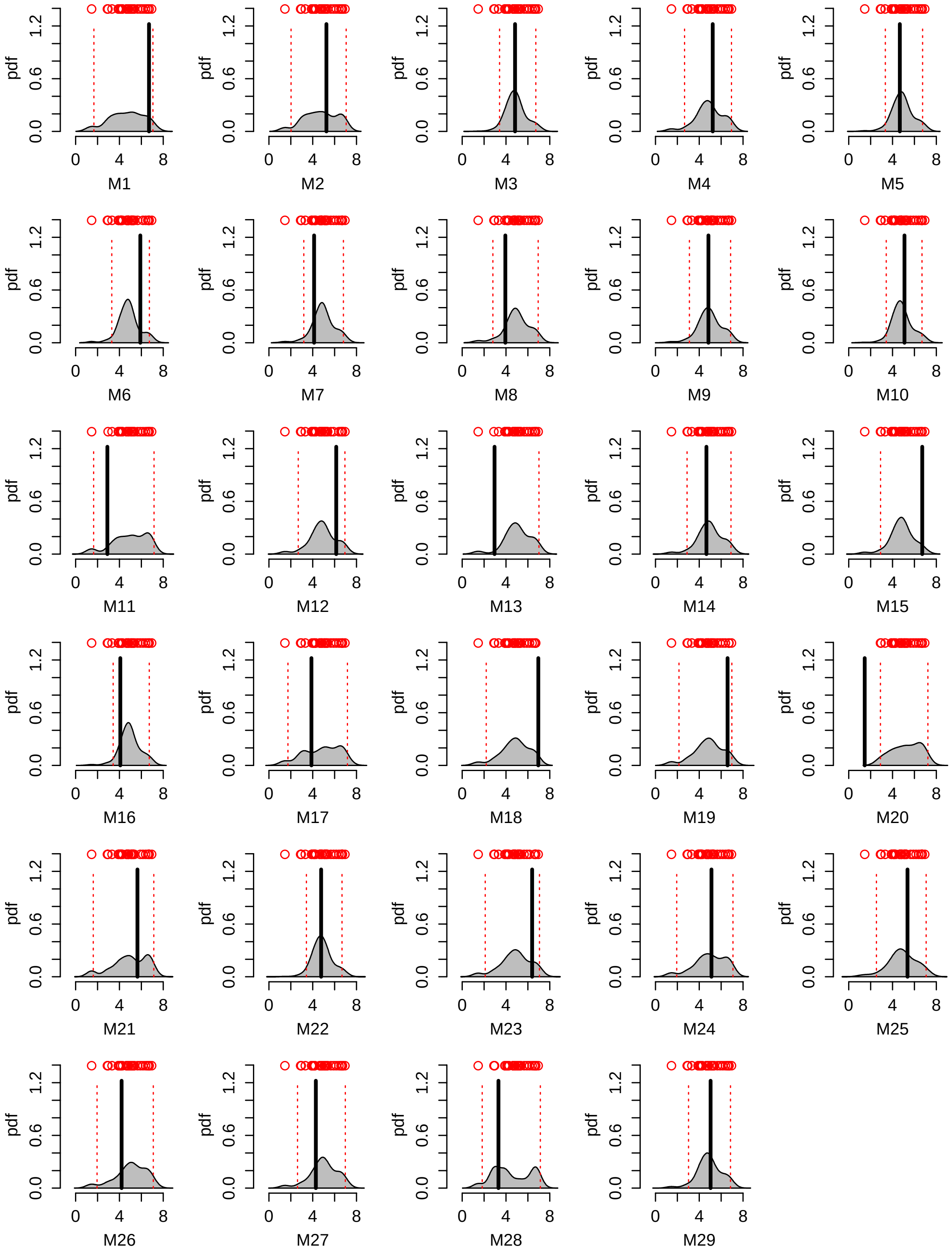

Fig P. Similar to Fig M, but for the Korea_temp "trend" experiment. The pdfs represent Korean JJA mean maximum temperature change from 1973-2005 to 2081-2100 [K] under the RCP8.5 emissions scenario.

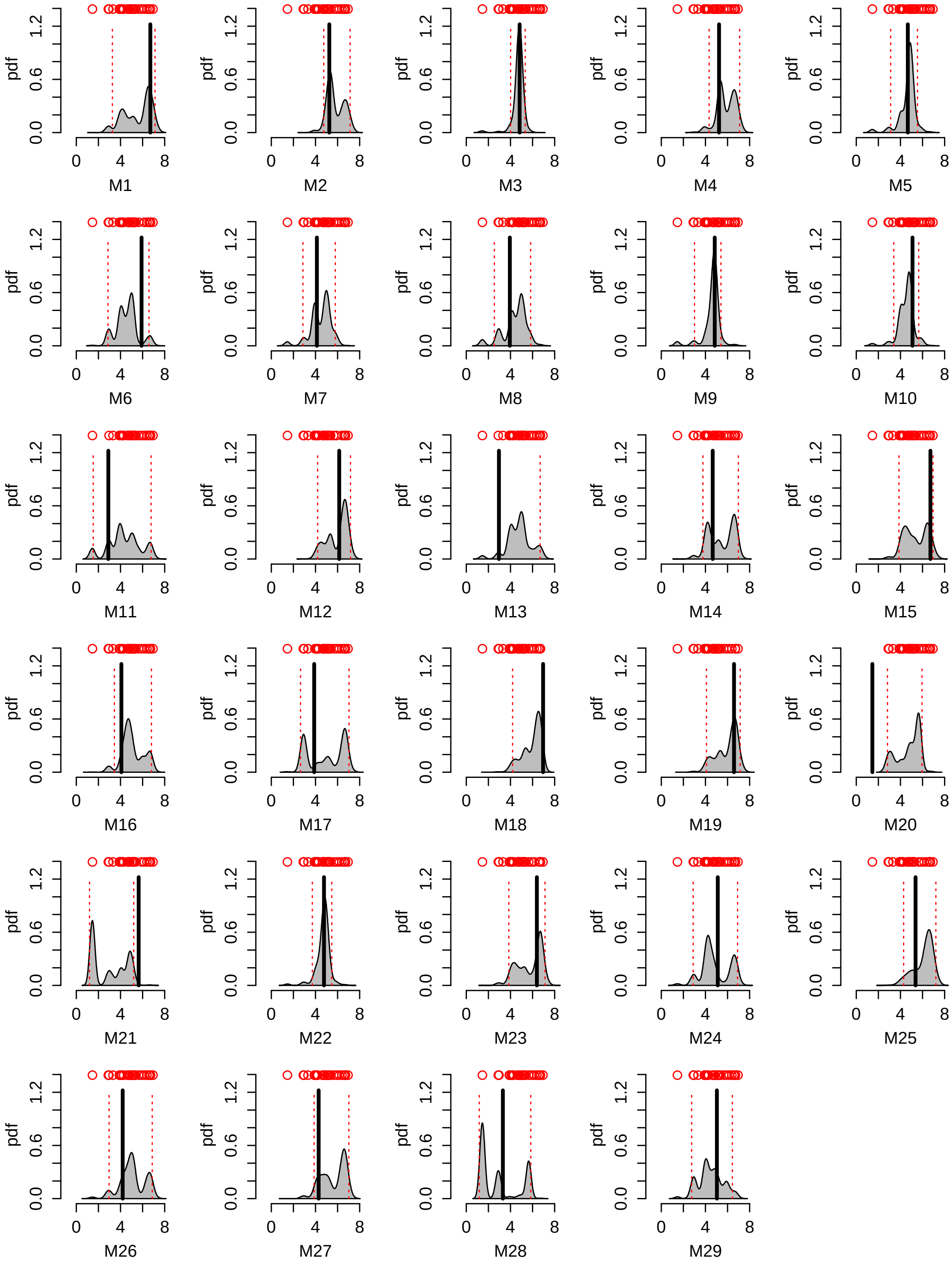

Fig Q. Similar to Fig O, but for the Korea_temp “trend+var” experiment.

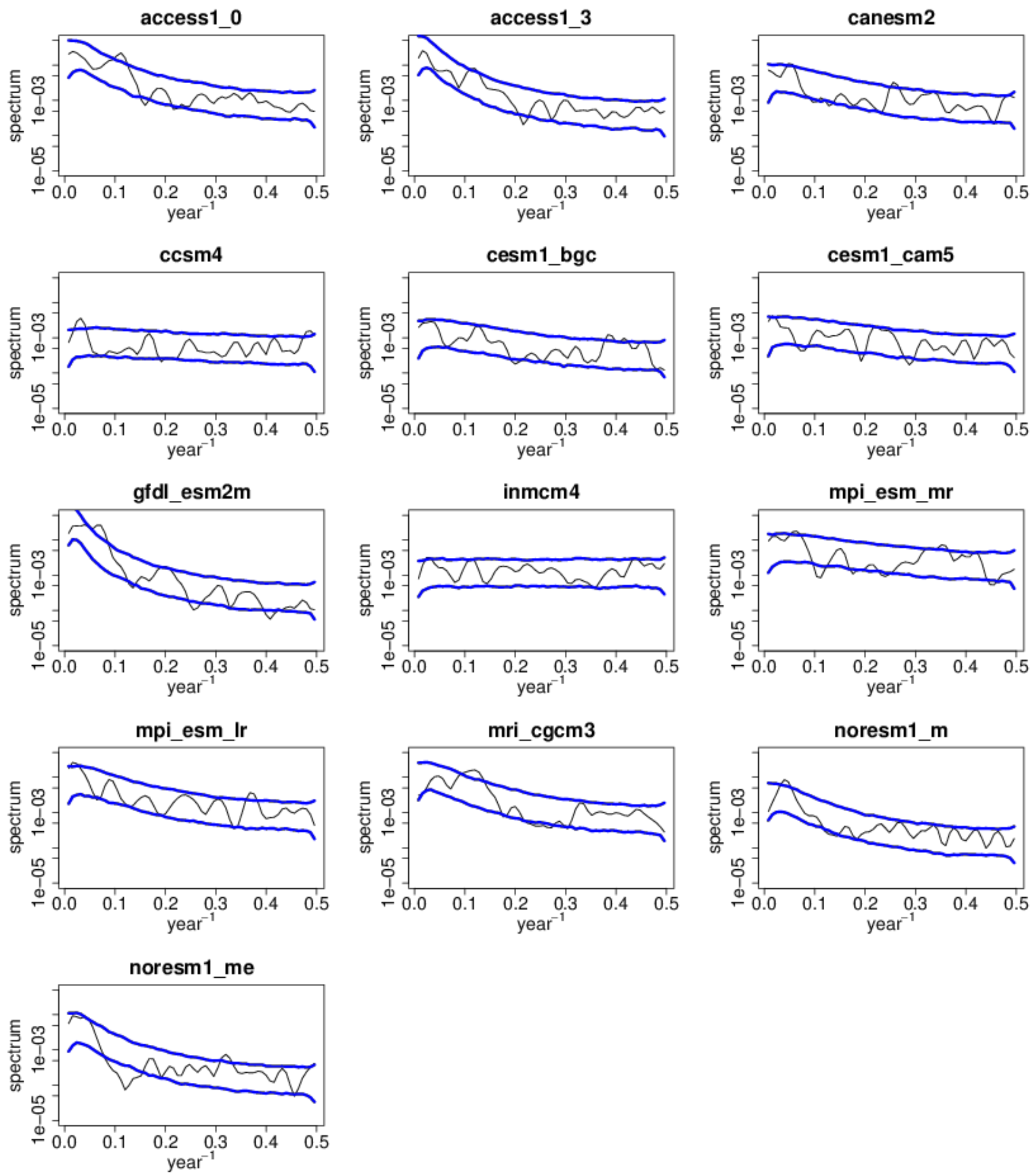

Fig R. Spectra plots for AMOC experiment model fluctuations for years 1880-2004. Blue lines: 90% confidence intervals for spectra of an AR1 process that was fit to modelled fluctuations, using 1000 realizations. *Y*-axis is logarithmic.

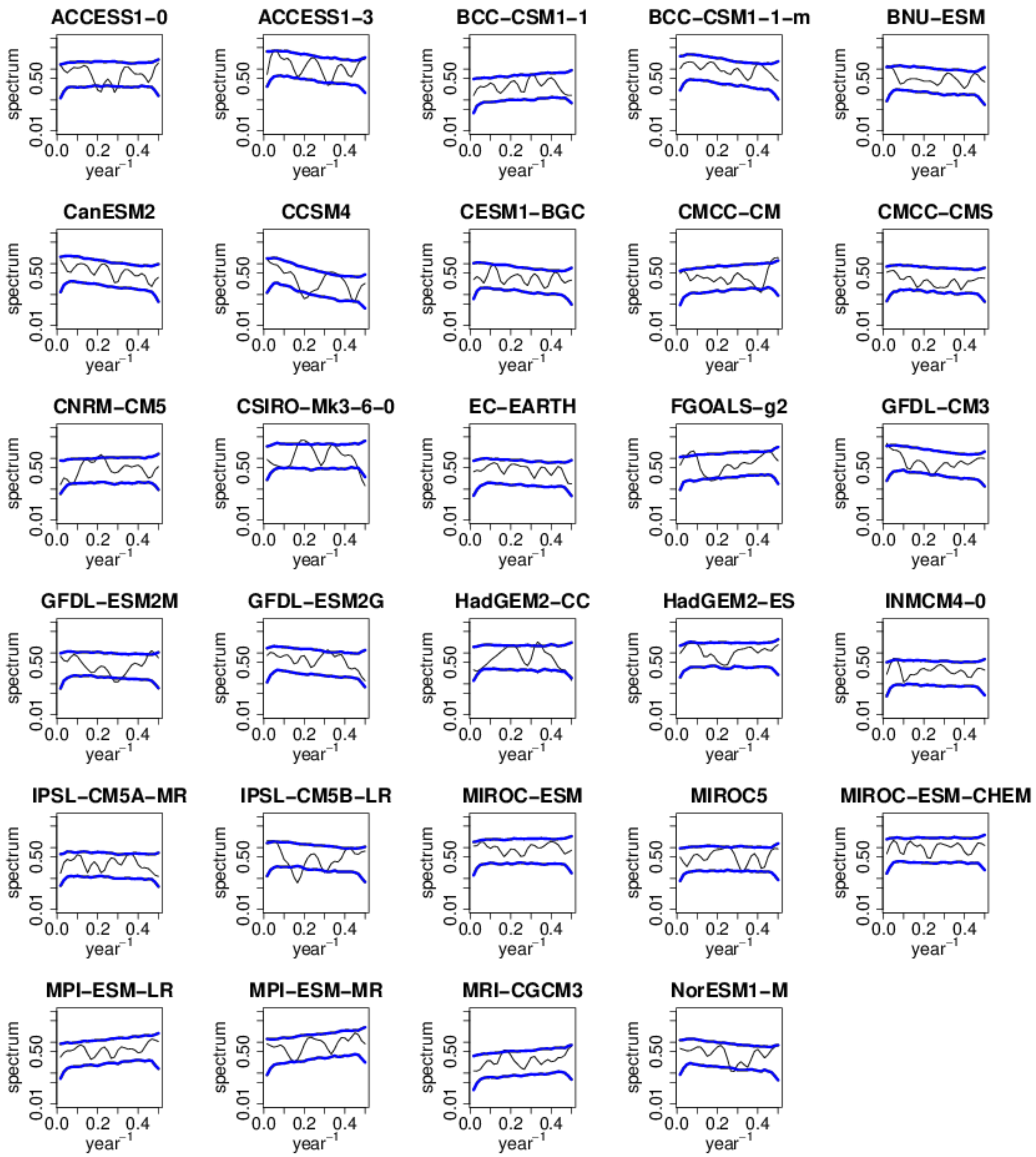

Fig S. Same as Fig Q but for Korea_temp_long experiment model fluctuations for years 1950-2005. *Y*-axis is logarithmic.

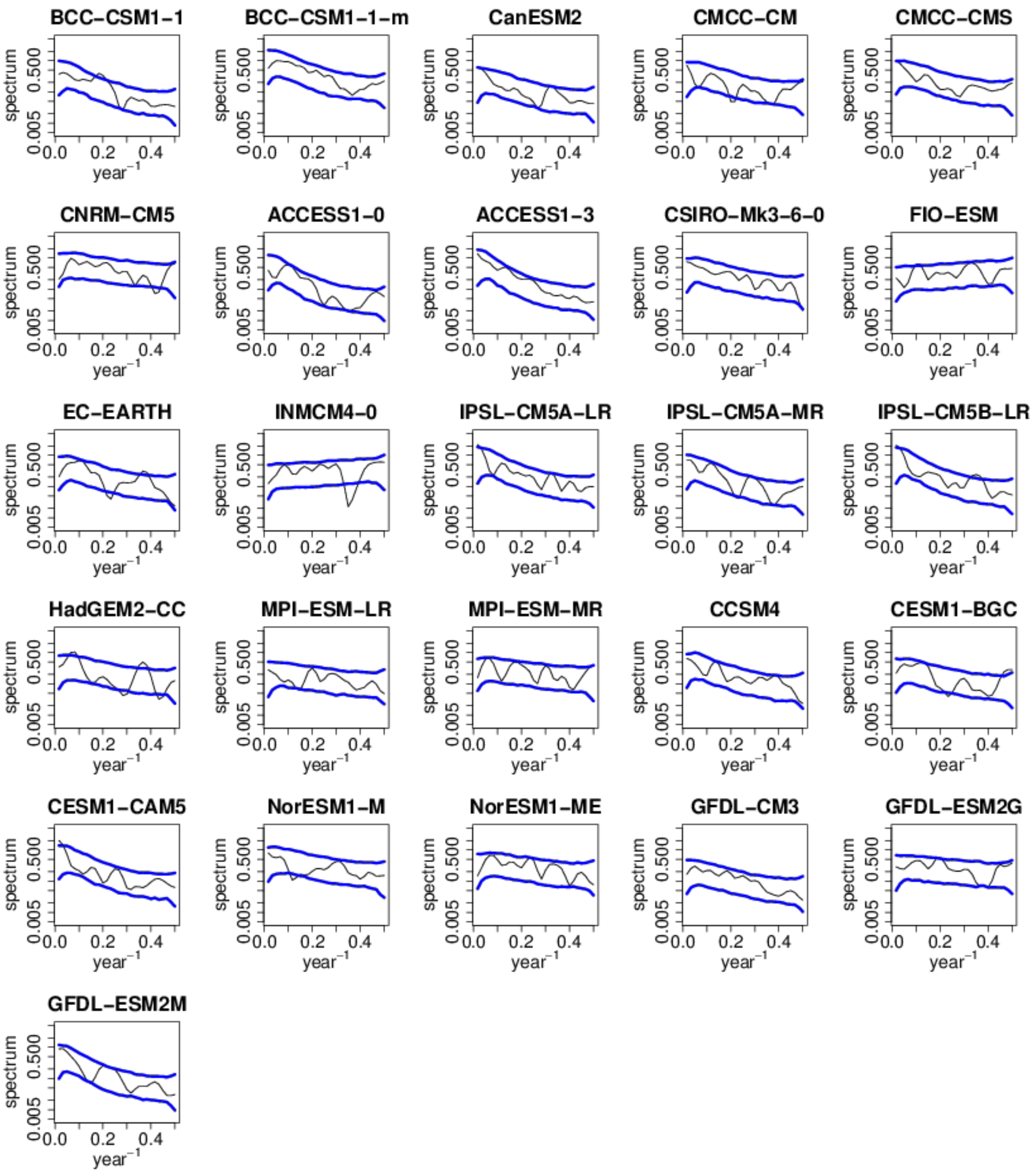

Fig T. Same as Fig Q but for Winter_SST_ experiment model fluctuations for years 1941-2000. *Y*-axis is logarithmic.

Model Number	Name	Modeling Centre
1	ACCESS1-0	CSIRO and BOM, Australia
2	ACCESS1-3	CSIRO and BOM, Australia
3	bcc-csm1-1	Beijing Climate Center & China Meteorological Administration, China
4	bcc-csm1.1-m	Beijing Climate Center & China Meteorological Administration, China
5	BNU-ESM	Beijing Normal University, China
6	CanESM2	Canadian Centre for Climate Modeling and Analysis, Canada
7	CCSM4	National Center for Atmospheric Research, USA
8	CESM1-BGC	National Science Foundation, Department of Energy, National Center for Atmospheric Research, USA
9	CMCC-CM	Euro-Mediterranean Centre on Climate Change, Italy
10	CMCC-CMS	Euro-Mediterranean Centre on Climate Change, Italy
11	CNRM-CM5	National Centre for Meteorological Research & European Centre for Research and Advanced Training in Scientific Computation, France
12	CSIRO-Mk3-6-0	Queensland Centre for Climate Change Excellence & CSIRO, Australia
13	EC-EARTH	EC-Earth consortium, Europe
14	FGOALS-g2	Institute of Atmospheric Physics, Chinese Academy of Sciences, China
15	GFDL-CM3	Geophysical Fluid Dynamics Laboratory, USA
16	GFDL-ESM2M	Geophysical Fluid Dynamics Laboratory, USA
17	GFDL-ESM2G	Geophysical Fluid Dynamics Laboratory, USA
18	HadGEM2-CC	Met Office Hadley Centre, UK
19	HadGEM2-ES	Met Office Hadley Centre, UK
20	inmcm4-0	Institute of Numerical Mathematics, Russia
21	IPSL-CM5A-MR	Institute Pierre Simon Laplace, France
22	IPSL-CM5B-LR	Institute Pierre Simon Laplace, France
23	MIROC-ESM	University of Tokyo, National Institute for Environmental Studies & Japan Agency for Marine-Earth Science and Technology, Japan
24	MIROC5	University of Tokyo, National Institute for Environmental Studies & Japan Agency for Marine-Earth Science and Technology, Japan
25	MIROC-ESM-CHEM	University of Tokyo, National Institute for Environmental Studies & Japan Agency for Marine-Earth Science and Technology, Japan
26	MPI-ESM-LR	Max Planck Institute for Meteorology (MPI-M), Germany
27	MPI-ESM-MR	Max Planck Institute for Meteorology (MPI-M), Germany
28	MRI-CGCM3	Meteorological Research Institute, Japan

29	NorESM1-M	Norwegian Climate Centre, Norway

Table A: Basic information about GCMs used for the Korea_temp and Korea_temp_long experiments.

Model Number	Name	Modeling Centre
1	bcc-csm1-1	Beijing Climate Center & China Meteorological Administration, China
2	bcc-csm1.1-m	Beijing Climate Center & China Meteorological Administration, China
3	CanESM2	Canadian Centre for Climate Modeling and Analysis, Canada
4	CMCC-CM	Euro-Mediterranean Centre on Climate Change, Italy
5	CMCC-CMS	Euro-Mediterranean Centre on Climate Change, Italy
6	CNRM-CM5	National Centre for Meteorological Research & European Centre for Research and Advanced Training in Scientific Computation, France
7	ACCESS1-0	CSIRO and BOM, Australia
8	ACCESS1-3	CSIRO and BOM, Australia
9	CSIRO-Mk3-6-0	Queensland Centre for Climate Change Excellence & CSIRO, Australia
10	FIO-ESM	The First Institute of Oceanography, SOA, China
11	EC-EARTH	EC-Earth consortium, Europe
12	inmcm4-0	Institute of Numerical Mathematics, Russia
13	IPSL-CM5A-LR	Institute Pierre Simon Laplace, France
14	IPSL-CM5A-MR	Institute Pierre Simon Laplace, France
15	IPSL-CM5B-LR	Institute Pierre Simon Laplace, France
16	HadGEM2-CC	Met Office Hadley Centre, UK
17	MPI-ESM-LR	Max Planck Institute for Meteorology (MPI-M), Germany
18	MPI-ESM-MR	Max Planck Institute for Meteorology (MPI-M), Germany
19	CCSM4	National Center for Atmospheric Research, USA
20	CESM1-BGC	National Science Foundation, Department of Energy, National Center for Atmospheric Research, USA
21	CESM1-CAM5	National Science Foundation, Department of Energy, National Center for Atmospheric Research, USA
22	NorESM1-M	Norwegian Climate Centre, Norway
23	NorESM1-ME	Norwegian Climate Centre, Norway
24	GFDL-CM3	Geophysical Fluid Dynamics Laboratory, USA
25	GFDL-ESM2G	Geophysical Fluid Dynamics Laboratory, USA
26	GFDL-ESM2M	Geophysical Fluid Dynamics Laboratory, USA

Table B. Basic information about GCMs used for the Winter_SST experiment.

References:

1. Olson R, An S-I, Fan Y, Evans JP, Caesar L. North Atlantic observations sharpen meridional overturning projections. Clim Dyn. 2017 Aug 23;1–18.
